# Supplementary material for: Localized carbon deposition enables trimming of photonic integrated circuits
Source: Nat Commun. 2026 May 21;17:4562. doi: 10.1038/s41467-026-73411-7 (PMC13194947; doi:10.1038/s41467-026-73411-7)
Supplement: Supplementary file 1 — Supplementary Information [file 41467_2026_73411_MOESM1_ESM.pdf]

Supplementary:

Localized Carbon Deposition Enables Trimming of Photonic Integrated Circuits

Rongyang Xu[1†], Zhongyu Tang[1†], Liam McRae[1], Akhil Varri[2], Frank Brückerhoff-Plückelmann[1], Xinyu Ma[1], Julian Rasmus Bankwitz[1], Julius Römer[1], Ravi Pradip[1], Qinlin Zhang[1], Lennart Meyer[1], Zhe Zhao[2], Jelle Dijkstra[1], Harish Bhaskaran[3], Rasmus R. Schröder[4], Wolfram H. P. Pernice[1], and Shabnam Taheriniya[1]**

[1] Kirchhoff-Institute for Physics, Heidelberg University, Im Neuenheimer Feld 227, 69120 Heidelberg, Germany

[2] Institute of Physics, University of Münster, Heisenbergstraße 11, 48149 Münster, Germany

[3] Department of Materials, University of Oxford, Parks Road, Oxford OX1 3PH, Oxfordshire, UK

[4] BioQuant, Heidelberg University, Im Neuenheimer Feld 267, 69120 Heidelberg, Germany

[†] These authors contributed equally: Rongyang Xu, Zhongyu Tang

*wolfram.pernice@kip.uni-heidelberg.de; shabnam.taheriniya@kip.uni-heidelberg.de

S1. FIB sample stage

The actual configuration of the FIB sample stage differs from the schematic diagram shown in the main text. In actual operation, the sample is tilted at an angle of 54°, allowing the obliquely mounted ion beam to strike the sample surface perpendicularly, while the electron beam is incident from the top to observe the sample, as shown in Supplementary Fig. 1.

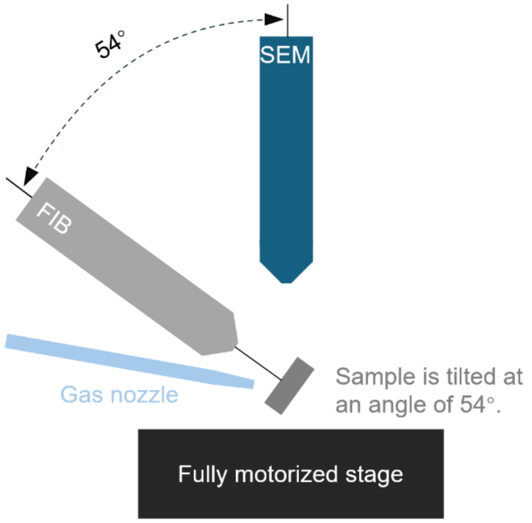

Supplementary Fig. 1 | Schematic of the actual FIB sample stage. The figure illustrates the configuration of the sample mounted on the stage during the FIB carbon deposition.

S2. Sample fabrication

Supplementary Fig. 2 shows the fabrication process of photonic structures prior to carbon deposition. First, the chip is cleaned with acetone and isopropanol, followed by spin-coating a 300-nm-thick negative-tone resist (Allresist AR-N 7520 series). Patterns are defined using electron-beam lithography, followed by development with MF-319 developer. The exposed resist serves as an etching mask. The silicon nitride regions not covered by the resist are etched using reactive ion etching (Oxford 80 RIE) with CHF_3 and O_2 gases. Finally, the resist mask is removed via oxygen plasma treatment.

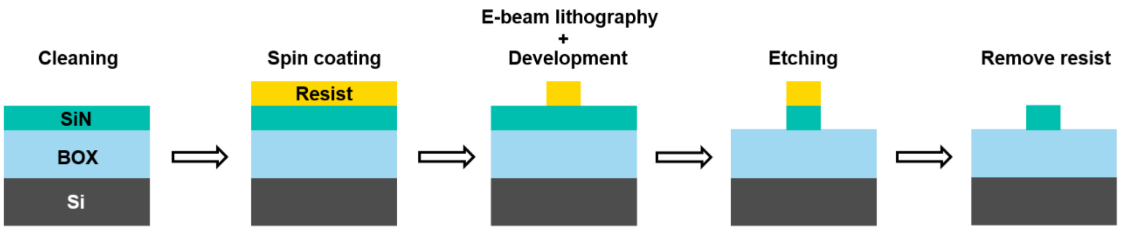

Supplementary Fig. 2 | Fabrication process of photonic structures. The figure outlines the key fabrication steps for the silicon nitride structures before the trimming process.

S3. FIB carbon deposition for cladded photonic structures

In practical applications, waveguides are often covered by a protective cladding layer to enable robust operation and future integration. It is therefore essential to assess the compatibility of the FIB carbon deposition with cladded structures. Supplementary Fig. 3a compares the effective refractive index change Δn_{eff} of carbon deposition for waveguides with and without a silica cladding. As confirmed in Supplementary Fig. 3b and 3c, the deposited carbon interacts less strongly with the waveguide mode as the cladding thickness increases and the carbon becomes more distant from the waveguide core, leading to a reduced Δn_{eff}. With a cladding thickness of 500 nm, the resulting Δn_{eff} is approximately one-quarter of the uncladded value.

For relatively thin claddings, the deposited carbon volume can be increased to compensate for the weakened interaction and maintain efficient trimming. However, when the cladding thickness approaches ~ 1000 nm, the interaction becomes too weak to enable meaningful tuning. In such cases, the FIB carbon deposition can be combined with FIB milling: Ga^+ ions can create nanoscale trenches within the SiO_2 cladding[1], reducing the distance between the deposited carbon and the waveguide core and thereby enhancing the interaction.

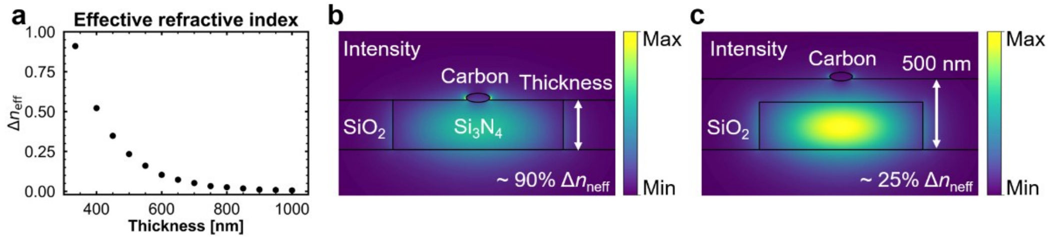

Supplementary Fig. 3 | Effect of carbon deposition for different cladding thicknesses. a Percentage change in n_{eff} for varying cladding thicknesses, normalized to the no-cladding case (Δn_{eff} = 0.008) using the same deposition volume. **b, c** Intensity distribution of carbon-trimmed waveguide with cladding thickness of 335 nm and 500 nm, respectively.

S4. Insertion loss and associated effective refractive index change

To evaluate the insertion loss per unit length and the corresponding Δn_{eff}, we fabricated a series of waveguide transmission test structures and Mach-Zehnder interferometers (MZIs), as shown in Supplementary Fig. 4. Carbon regions with lengths ranging from 50 to 200 μm, in steps of 50 μm, were deposited under identical conditions across all devices.

The insertion loss was extracted using the waveguide transmission test structures by first normalizing the output of the trimmed arm to the reference arm within the same device, and then comparing the normalized transmission before and after deposition. The waveguide transmission test structures yield a unit-length loss of 78 dB cm^{-1}, while the MZIs fabricated in the same batch give a similar value of 74 dB cm^{-1} for the same deposition lengths, as shown in Supplementary Fig. 5. The close agreement between these measurements confirms the reliability of the extracted

loss, whose averaged value of 76 dB cm^{-1} is reported in the main text. The Δn_{eff} was subsequently determined from the spectral shifts of these MZIs, giving an average value of approximately 0.013.

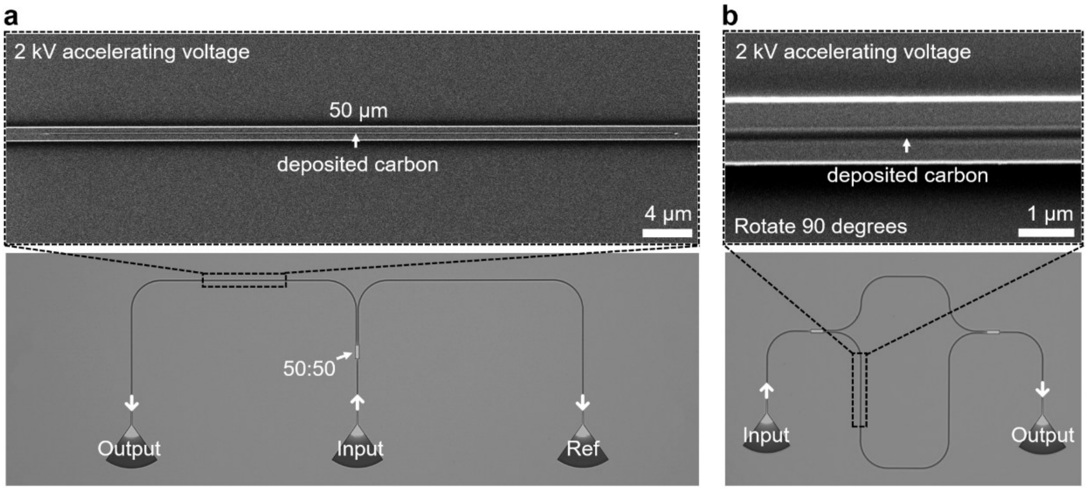

Supplementary Fig. 4 | Images of representative devices after trimming. a Optical microscope and SEM views of the waveguide transmission test structure. **b** Optical microscope and SEM views of the MZI used to extract the loss and the effective refractive index change.

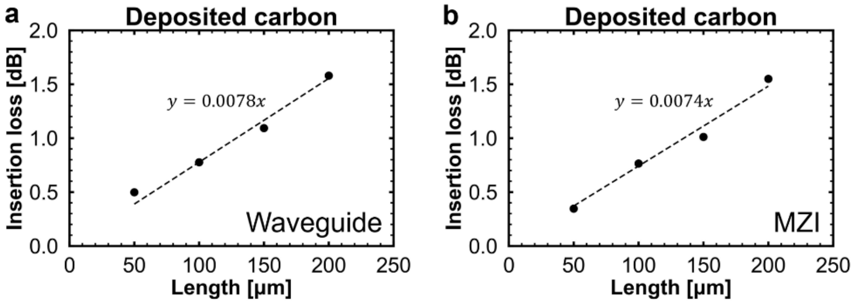

Supplementary Fig. 5 | Insertion loss of carbon deposition obtained from representative devices. a Extracted insertion loss for deposited carbon of different lengths on waveguide transmission test structures. **b** Extracted insertion loss obtained from the MZIs. Carbon deposition on both types of structures was carried out under identical deposition conditions.

S5. Transmission after thermal treatment

Because thermal treatment can accelerate the stabilization of the optical response, we measured the transmission of multiple devices after cumulative hot-plate heating at 200°C for 10, 20, 30, and 50 minutes. The transmission of all devices shows similar trends over time. Supplementary Fig. 6 shows the transmission of a TE_1 mode asymmetric directional coupler (A-DC). The insertion loss decreases from 9.21 dB to 1.62 dB, as measured 5 days after carbon deposition. Two days later, the A-DC was placed on a hot plate for thermal treatment. During the first 20 minutes of cumulative heating, A gradual decrease in transmission was observed during the first 20 minutes, after which the device reached a stable level. Although a further decrease was seen at 30 minutes, this is likely

due to measurement errors, as the transmission after 50 minutes returned to the same level as that at 20 minutes.

In addition, ring resonators were used to further verify the effect of thermal treatment after carbon deposition on the stability of the optical response. Supplementary Fig. 7a shows a focused ion beam image of a ring resonator after carbon deposition. The response of the ring resonator was first measured before carbon deposition. Three days later, the same device was re-measured, both before and after a 30-minute thermal treatment. A red shift of 1.6 nm was observed, which decreased to 1 nm after the treatment. Nearly four weeks later, the ring resonator was measured once more, and the resonant peak remained unchanged, as shown in the zoomed-in image in Supplementary Fig. 7b, confirming the stability of the optical response over time.

To quantify the trimming performance, we evaluate the as-trimmed resonance peak shift of 1.6 nm. With a free-spectral range of 6.5 nm, this corresponds to a phase shift of approximately 0.5 π. The relatively thick deposited carbon in this ring resonator leads to an extracted local Δn_{eff} of 0.038. The carbon deposition introduces an additional round-trip loss of 0.2 dB, corresponding to 0.4 dB π^{-1}, which is comparable to the loss-per-π extracted from the MZI in section S7.

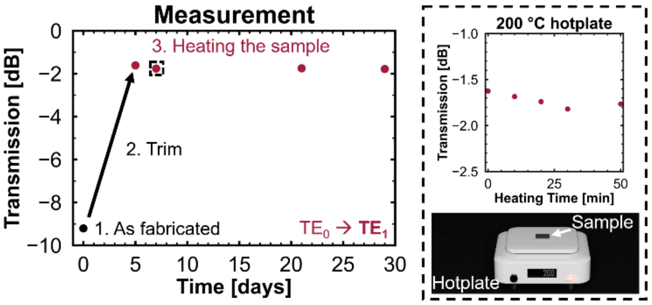

Supplementary Fig. 6 | Change in optical response after deposition. Transmission of the TE_1 mode A-DC after carbon deposition changes with time. Optical response can be stabilized by thermal treatment on a hot plate at 200 °C for more than 20 minutes.

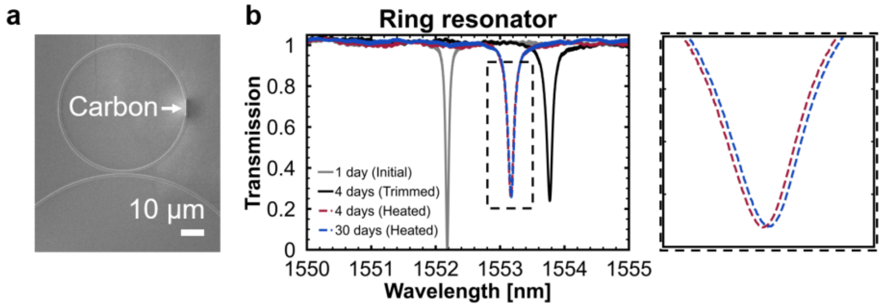

Supplementary Fig. 7 | Carbon deposition on a ring resonator. a A focused ion beam image of a ring resonator after carbon deposition. **b** Transmission peaks of the ring resonator before and after carbon deposition and thermal treatment. The day on which carbon deposition was performed was defined as day one.

S6. Layout of the photonic crossbar array with trimmed asymmetric directional couplers

The optical microscope image of the photonic crossbar array presented in the main text is shown in Supplementary Fig. 8. The structure comprises two typical 9×2 photonic crossbar arrays and two A-DCs that combine the output light from the two arrays with minimal loss via mode-division multiplexing. Owing to the flexibility of the FIB carbon deposition, the trimming can be performed directly on the fabricated chip without requiring any predetermined auxiliary structures.

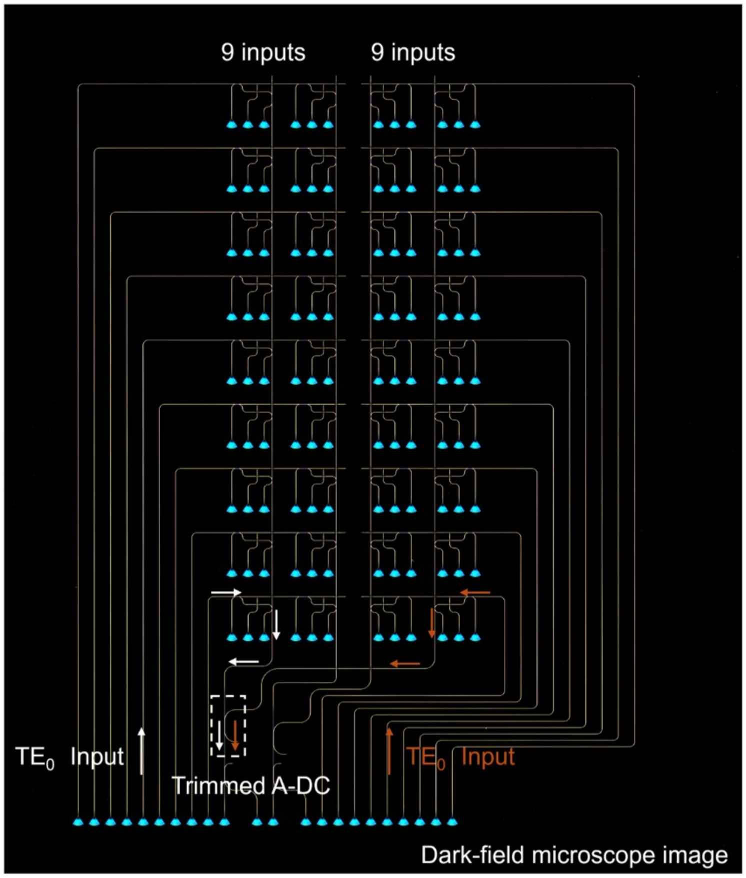

Supplementary Fig. 8 | Carbon deposition on a photonic crossbar array. Optical microscope image of the fabricated device.

S7. Trimming of directional couplers and Mach-Zehnder interferometers

The proposed trimming method is not limited to the A-DC in the main text; it can also be applied to other commonly used devices, such as directional couplers and MZIs. To illustrate this, we fabricated a directional coupler designed to exhibit a 0.5 coupling ratio. Due to a slightly narrower fabricated gap, the actual coupling was stronger than intended, resulting in a measured ratio of 0.75. To correct this deviation, we deposited an approximately 8-μm-long region of carbon on the waveguide, corresponding to roughly one-third of the effective coupling length, as shown in Supplementary Fig. 9a and 9b. To introduce a sufficiently large effective-index contrast between the two waveguide modes and ensure efficient coupling suppression, carbon was deposited twice

at nearly the same location. After trimming, the coupling ratio was reduced from 0.75 to the intended value of 0.5, as illustrated in Supplementary Fig. 9c.

We next applied the method to MZIs, which offer a convenient platform for benchmarking trimming performance because their interference fringes provide a direct measure of phase changes. A 50-μm-long carbon was deposited on one arm of the fabricated MZI, as shown in Supplementary Fig. 9d and 9e. As seen in Supplementary Fig. 9f, the deposition produces a near-π phase shift, while the corresponding insertion loss of 0.35 dB is extracted from the extinction ratio between the maximum and minimum transmission. This yields a trimming efficiency of 0.35 dB π^{-1}, which we benchmark against representative non-volatile trimming methods in Table 1 of the main text.

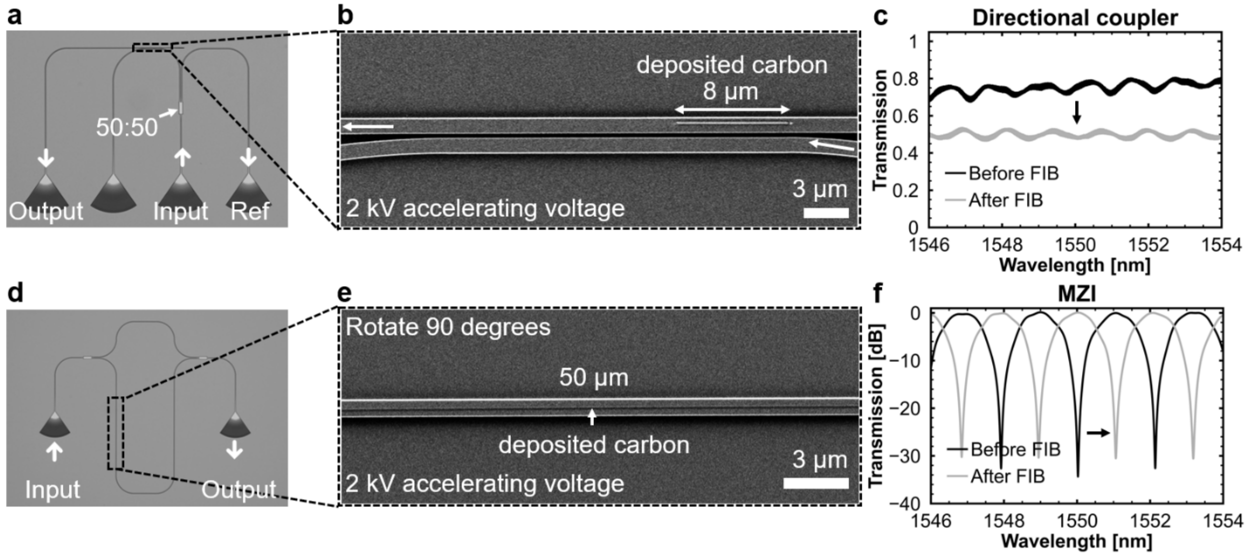

Supplementary Fig. 9 | Application of FIB carbon deposition in directional couplers and MZIs. a, b Optical microscope image and SEM image of the trimmed directional coupler with a coupling length of 25 μm. **c** The transmission spectra of the directional coupler before and after FIB carbon deposition. **d, e** Optical microscope image and SEM image of the trimmed MZI with a carbon-deposition length of 50 μm. **f** Transmission spectra of the trimmed MZI after carbon deposition, showing a near-π phase shift.

Reference:

1. Menard, L. D. & Ramsey, J. M. Fabrication of Sub-5 nm Nanochannels in Insulating Substrates Using Focused Ion Beam Milling. *Nano Lett.* **11**, 512–517 (2011).
